# Accumulation of starch in Zn-deficient rice

**DOI:** 10.1186/1939-8433-5-9

**Published:** 2012-04-06

**Authors:** Motofumi Suzuki, Khurram Bashir, Haruhiko Inoue, Michiko Takahashi, Hiromi Nakanishi, Naoko K Nishizawa

**Affiliations:** 1 Graduate School of Agricultural and Life Sciences, The University of Tokyo, 1-1-1 Yayoi, Bunkyo-ku, Tokyo, 113-8657 Japan; 2 Research Institute for Bioresources and Biotechnology, Ishikawa Prefectural University, 1-308 Suematsu, Nonoichi-shi, Ishikawa, 921-8836 Japan

**Keywords:** Microarray, Rice, RNase, Starch accumulation, Zinc, Zinc deficiency

## Abstract

Zinc (Zn) is an essential micronutrient for living organisms, and understanding the molecular mechanisms of Zn deficiency may help to develop strategies to mitigate this problem. Microarray analysis of Zn deficient rice revealed the up-regulation of several genes involved in Zn transport. Moreover many genes involved in starch synthesis/transport were up-regulated by Zn deficiency in rice roots and shoots. Furthermore, starch granules were detected mainly in the cortical cells of these tissues. The gene encoding inactive RNase was much more highly transcribed than those encoding active RNases. Although the level of RNA degradation in a crude extract of Zn-deficient shoots was higher than that of Zn-sufficient shoots, addition of Zn significantly reduced the level of degradation. These results indicate that RNA degradation could be regulated by the amount of Zn in the cell, and that the tolerance of rice plants to low levels of Zn is promoted by the accumulation of starch and inactive RNase.

**Electronic supplementary material:**

The online version of this article (doi:10.1186/1939-8433-5-9) contains supplementary material, which is available to authorized users.

## Background

Zinc (Zn) is an essential micronutrient for almost all organisms, and its deficiency represents a serious nutritional problem in humans and plants. Zn is a non redox active element and serves as a cofactor for large number of enzymes involved in DNA transcription, protein, nucleic acid, carbohydrate, and lipid metabolism (Ishimaru et al. [2011]; Broadley et al. [2007]; Marschner [1995]). For example, DNA and RNA polymerases require Zn as a cofactor, and Zn is also essential for cell division. Indeed, Zn concentration in plant meristems is much higher than in other tissues (Kitagishi & Obata [1986]). Zn also plays a role in the structural stability of certain proteins, such as those containing Zn-finger domains, a dominant feature of many transcription factors. Genomic research in *Arabidopsis* revealed that roughly 22% of transcription factors contain a Zn-finger domain (Riechmann et al. [2000]); therefore, Zn may be important in regulating gene expression.

Many regions of the world, particularly those with calcareous soils, lack sufficient Zn, resulting in poor plant growth. Therefore, the mechanism for tolerance to Zn deficiency should be elucidated to mitigate Zn deficiency. Several mechanisms have been investigated to clarify the physiological basis of differential Zn efficiency among wheat genotypes. For example, cultivars tolerant to Zn deficiency secrete higher amounts of mugineic acid family phytosiderophores (MAs) than intolerant cultivars (Cakmak et al. [1994]; Walter et al. [1994]; Zhang et al. [1989]; Suzuki et al. [2006]; Cakmak et al. [1996]). MAs are synthesized from L-methionine (Mori & Nishizawa [1987]; Shojima et al. [1990]; Ma et al. [1995]; Ma et al. [1999]). Nicotianamine (NA) synthase (NAS) transforms three molecules of S-adenosyl-L-methionine to one molecule of NA, and NA aminotransferase (NAAT) catalyzes the amino transfer of NA. The keto form is subsequently reduced to 2′-deoxymugineic acid (DMA) by DMA synthase (DMAS) (Bashir et al. [2006]; Bashir & Nishizawa [2006]; Bashir et al. [2010]). Zn-DMA, is suggested to be preferred over Zn^2+^ for uptake in barley roots (Suzuki et al. [2006]), while rice roots absorb less Zn-DMA compared to Zn^2+^ (Suzuki et al. [2008]). Similarly the secretion of MAs increases in Zn deficient barley, while it decreases in rice (Suzuki et al. [2006]; Suzuki et al. [2008]). Despite this, Zn-DMA is suggested to be the preferred form for the long distance transport in rice (Suzuki et al. [2008]). Difference in DMA secretion is suggested to increase tolerance in rice (Widodo et al. [2010]). Moreover, a modelling study also proposed a strong correlation between DMA secretion and rooting density, and suggested a role of DMA for Zn absorption in rice (Ptashnyk et al. [2011]). The identification of Zn-NA complexes in rice phloem sap also suggests that NA performs a significant role in Zn transport (Nishiyama et al. [2012]). Zn uptake by roots, and translocation within the plant, are associated with Zn efficiency (Rengel & Graham [1996]), while the availability of Zn at the cellular level is also suggested to be related to Zn efficiency (Cakmak et al. [1997]). Moreover the expression level or activity of enzymes requiring Zn is shown to be different between Zn-efficient and Zn-inefficient cultivars (Hacisalihoglu et al. [2003]). Partitioning of carbohydrates also shows correlation with tolerance to Zn-deficiency stress (Pearson & Rengel [1997]). Rice cultivars differing in Zn efficiency have been used for physiological and genetic analyses of tolerance to Zn deficiency (Gao et al. [2005]; Hajiboland et al. [2005]; Hoffland et al. [2006]; Wissuwa et al. [2006]). Among graminaceous plants, rice is highly sensitive to Zn-deficient stress; thus, we investigated the physiological change and gene expression pattern in rice during Zn deficiency. Profiling of the genes involved in Zn-deficient stress is critical to elucidate the mechanisms of tolerance to or damage by Zn deficiency. We performed a microarray analysis with Zn-deficient and Zn-sufficient rice to identify genes whose expression increases in response to Zn deficiency. Based on our combined expression and phenotypic analyses, we demonstrated that besides up-regulation of genes involved in Zn uptake and transport, Zn deficiency enhances starch accumulation both in roots and shoots, and that Zn deficiency induces the expression of a gene encoding putative inactive RNase, which may function as vegetative storage protein. Further, the level of RNA degradation was increased by Zn deficiency.

## Results

### Up-regulation of genes involved in Zn transport

Microarray analysis revealed that the genes involved in Zn uptake and transport were up-regulated, both in roots and shoots of Zn deficient rice. The expression of *OsNAS1* particularly increased in rice shoots (Table 1). *OsNAS1* is reported to be up-regulated by iron deficiency in root and shoot tissue (Inoue et al. [2003]; Bashir et al. [2011]; Ishimaru et al. [2009]). Moreover, the expression of *OsNAS3* was also upregulated in root and shoot tissue. The expression of *OsNAS3* is not regulated by Fe deficiency and is reported to be up-regulated by Zn deficiency (Suzuki et al. [2008]). As NA was not detected in Zn deficient rice shoot, the NA catalyzed by OsNAS3 may be transformed to DMA (Suzuki et al. [2008]). The up-regulation of *OsNAAT1* supports this hypothesis. Besides this the expression of Zn transporters, *OsZIP4* *OsZIP5* and *OsZIP8* increased significantly in Zn deficient roots and shoots, while the expression of *OsZIP7* increased in shoot tissue (Table 1). *OsZIP4* and *OsZIP5* are plasma membrane Zn transporters induced by Zn deficiency (Lee et al. [2010]; Yang et al. [2009]; Ishimaru et al. [2005]). Moreover, the expression of *OsHMA1* was also induced by Zn deficiency in shoot tissue. OsHMA1 is supposed to be involved in Zn transport (Williams & Mills [2005]).

**Table 1:** **Microarray analysis of genes involved in Zn transport**

Accession No.	Gene	Ratio (−Zn/+Zn)	
		Root	Shoot
AK112069	*OsNAS1*	0.9 ± 0.4	17.8 ± 0.2
AK112011	*OsNAS2*	0.9 ± 0.4	1.2 ± 0.1
AK070656	*OsNAS3*	4.1 ± 0.4	2.7 ± 0.4
AK108576	*OsNAAT1*	10.6 ± 1.0	6.0 ± 0.6
AK107681	*OsIRT1*	1.0 ± 0.1	1.0 ± 0.1
AY302058	*OsZIP1*	1.0 ± 0.1	1.2 ± 0.0
AK121551	*OsZIP2*	1.1 ± 0.2	1.2 ± 0.2
AK069804	*OsZIP3*	0.7 ± 0.3	1.1 ± 0.2
AK105258	*OsZIP4*	11.2 ± 0.1	55.2 ± 4.1
AK070864	*OsZIP5*	3.8 ± 0.3	8.1 ± 0.3
AK103730	*OsZIP6*	1.1 ± 0.1	1.2 ± 0.1
AK071272	*OsZIP7*	1.8 ± 0.1	2.3 ± 0.0
AY327038	*OsZIP8*	6.0 ± 0.3	8.9 ± 1.3
Os05g0472400	*OsZIP9*	1.5 ± 0.6	1.0 ± 0.1
Os06g0566300	*OsZIP10*	1.0 ± 0.1	1.0 ± 0.0
Os05g0198400	*Zinc transporter*	2.3 ± 0.0	5.8 ± 0.1
Os06g0690700	*OsHMA1*	1.7 ± 0.5	7.4 ± 2.0

### Starch accumulation in Zn-deficient roots and shoots

As shown in Table 2, genes encoding starch synthase, ADP-glucose pyrophosphorylase (AGPase) large subunit, AGPase small subunit, α-1,4-glucan branching enzyme, and phosphoglucomutase, which are thought to be involved in starch metabolism, were up-regulated by Zn deficiency. Sucrose synthase, which reversibly converts sucrose to ADP-glucose, was up-regulated in Zn-deficient shoots and slightly up-regulated in roots, while α-1,4-glucan phosphorylase, which catalyzes the phospholysis of a linear glucan chain to reversibly yield glucose 1-phosphate (Smith et al. [2003]), was also up-regulated. Although multiple pathways may exist for starch synthesis (Baroja-Fernández et al. [2004]), the above enzymes may be sufficient for starch synthesis. Moreover, several genes involved in sugar transporter were up-regulated especially in shoots. The expression of these genes increased more significantly, when plants were subjected to Zn deficiency and analysed by 22 K microarray analysis ( Additional file 1: Table S1).

**Table 2:** **Microarray analysis of genes involved in carbohydrate metabolism and transport in rice**

Accession No.	Putative gene identification	Ratio (−Zn/+Zn)	
		Root	Shoot
**Starch metabolism and transport**			
AK102058	*Starch synthase*	3.5 ± 0.4	2.5 ± 0.8
AK100910	*AGPase large subunit*	2.4 ± 0.2	2.7 ± 0.3
AK073146	*AGPase small subunit*	2.0 ± 0.4	2.2 ± 0.2
AK068920	*α-1,4-glucan branching enzyme*	1.6 ± 0.2	2.9 ± 0.4
AK065121	*α-1,4-glucan branching enzyme*	1.4 ± 0.2	2.1 ± 0.2
AK064893	*Phosphoglucomtase*	3.7 ± 0.3	1.8 ± 0.0
AK099406	*Sucrose synthase*	1.5 ± 0.1	1.9 ± 0.1
AK063766	*α-1,4-glucan phosphorylase*	1.5 ± 0.1	2.2 ± 0.2
AK103367	*α-1,4-glucan phosphorylase*	2.2 ± 0.1	1.8 ± 0.2
AK073216	*Sorbitol transporter*	1.4 ± 0.1	2.7 ± 0.2
AK073967	*Hexose transporter*	1.7 ± 0.3	1.8 ± 0.3
AK073216	*Sugar transporter*	1.4 ± 0.1	2.7 ± 0.2
AK060577	*Glucose-6-phosphate translocator*	1.3 ± 0.2	2.1 ± 0.2
AK059423	*Sugar transporter*	0.8 ± 0.1	2.0 ± 0.4
AK069202	*Glucose transporter*	1.1 ± 0.1	2.2 ± 0.1
AK103915	*Sugar transporter protein*	2.4 ± 0.5	2.0 ± 0.1

**Figure 1:**
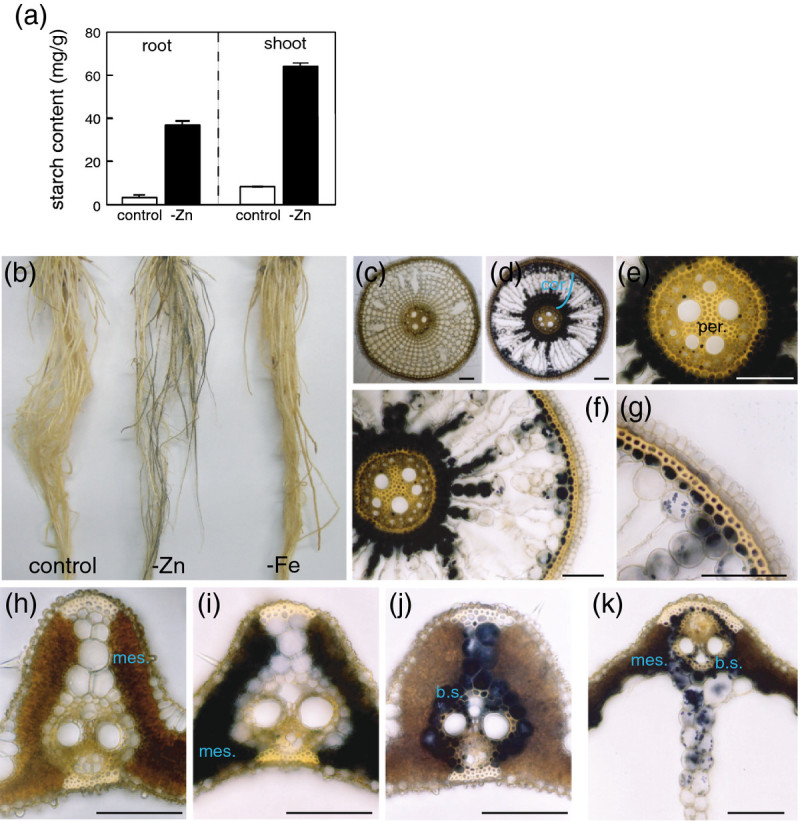
**Quantification of starch (a) and localization of starch granules in Zn-deficient and Zn-sufficient (control) plants by iodine staining (b–k). (b)** Zn- and Fe-sufficient roots (control); Zn-deficient roots (−Zn) and Fe-deficient roots (−Fe), **(c)** control roots, **(d–g)** Zn-deficient roots, **(h)** medium vein of old control leaf, **(i)** medium vein of Zn-deficient newest mature leaf, **(j)** medium vein of Zn-deficient old leaf, **(k)** large vein of Zn-deficient old leaf. Bar = 100 μm. cor.; cortex, mes.; mesophyll cells, b.s.; bundle sheath cells.

The starch content was higher in both roots and shoots grown under Zn-deficient conditions (Figure 1a), corresponding to the gene expression pattern revealed by our microarray analysis. Iodine–starch reaction staining revealed starch accumulation in Zn-deficient roots, but not in control roots or Fe-deficient roots (Figure 1b–d). In Zn-deficient roots, several starch granules were detected in the cortex, especially in the cells close to the sclerenchyma and endodermis (Figure 1d, f, g), and additional granules were observed in the pericycle (Figure 1e). In shoots, starch granules were mainly found in the mesophyll cells of the control plants (Figure 1h); however, in Zn-deficient shoots, the location of the starch granules was different among the leaves within a plant. Starch accumulated in the mesophyll cells of the youngest mature leaves of Zn-deficient rice (Figure 1i). When the youngest leaf was immature, starch accumulated in the bundle sheath cells of older leaves (Figure 1j). At the large vein of old leaves, starch granules were observed both in the mesophyll cells and in the bundle sheath cells (Figure 1k).

### Zn-deficient rice strongly expresses inactive RNase

**Figure 2:**
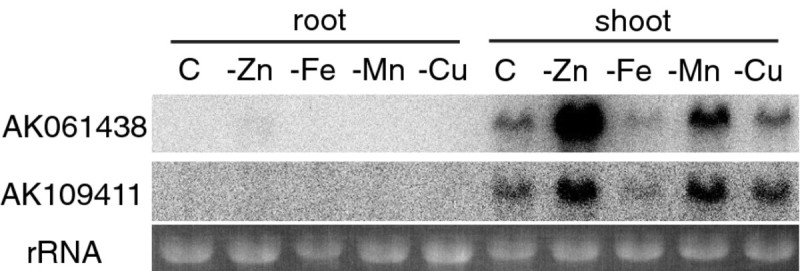
**The expression pattern of the genes encoding S-like RNases predicted to lack RNAse activity.** Each lane contained 10 μg of total RNA. C, control; -Zn, grown with a low Zn supply; -Fe, grown with a low Fe supply; -Mn, grown with a low Mn supply; -Cu, grown with a low Cu supply.

Among the 27,800 genes analyzed, *Os09g0537700* (AK061438; OsRNS4), which is predicted to encode S-like RNase, showed the highest induction ratio in roots (Table 3). This gene was also up-regulated in Zn-deficient shoots. Other genes encoded putative RNases, were not significantly up-regulated by Zn deficiency. In rice eight genes for S-like RNase have been described (MacIntosh et al. [2010]) and among them only six were included in our microarray experiment. In addition, the transcript levels of AK061438 in Zn-deficient shoots were much higher than those of the other RNases. Northern blot analysis revealed that the expression of two S-like RNases increased in Zn-deficient shoots, but not in Fe-deficient or Cu-deficient shoots (Figure 2); Mn deficiency slightly induced expression. Although the expression ratio of AK061438 in Zn-deficient roots was very high, the transcript level in roots was much lower than in shoots. Northern blot analysis as well as 22 K microarray analysis of rice plants indicated that expression of *OsRNS5* also increases by Zn deficiency (Figure 2 & Additional file 1: Table S2).

**Table 3:** **Microarray profile of genes encoding RNase in rice**

Accession No.	Gene	Ratio (−Zn/+Zn)		Putative enzyme activity
		Roots	Shoots	
AK060320	*OsRNS1*	0.6 ± 0.1	1.1 ± 0.3	Yes
AK105061	*OsRNS2*	1.1 ± 0.1	1.4 ± 0.1	Yes
AK058502	*OsRNS3*	1.2 ± 0.3	1.2 ± 0.3	Yes
AK061438	*OsRNS4*	29.2 ± 10.7	5.7 ± 0.41	No
AK109411	*OsRNS5*	1.0 ± 0.0	1.2 ± 0.1	No
AK060320	*OsRNS6*	0.6 ± 0.1	1.1 ± 0.3	Yes

**Figure 3:**
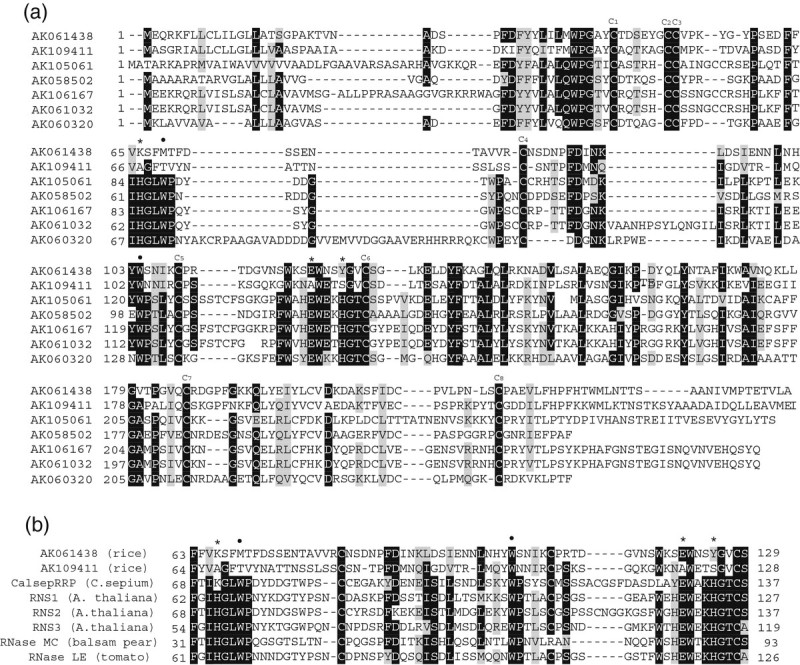
**Comparison of the amino acid sequences of RNase and RNase-related proteins in plants. (a)** All of the amino acid sequences predicted from the rice full-length cDNA database. **(b)** Partial amino acid sequences from *C. sepium* (CalsepRRP), *A. thaliana* (RNS1, RNS2, and RNS3), *M. charantia* (RNase MC), and *L. esculentum* (RNase LE). Gaps were introduced to maximize the homologies. Identical or similar residues are boxed in black and grey, respectively. The three charged residues involved in the active site of RNase are indicated by asterisks, and the two aromatic residues, which presumably maintain the conformational stability of the site, are indicated by a black circle. The sequences of the eight conserved cysteine residues in rice are indicated by C1–C8.

An alignment of the amino acid sequences of the RNases is shown in Figure 3a. All of the RNases contain eight conserved cysteine residues involved in the three-dimensional formation of RNase (Rabijns et al. [2002]); however, the amino acid sequences encoded by AK061438 and AK109411 lack two histidine residues (replaced by Lys66 and Tyr125 in AK061438, and by Ala67 and Ser125 in AK109411) that are essential for RNA degradation (Kurihara et al. [1996]). In addition, the glutamate residue, which is also essential for RNA degradation, is not conserved in AK109411 (replaced by Ala121). These histidine and glutamate residues are conserved in RNases in other plant species (Figure 3b), excluding CalsepRRP, which is an inactive RNase because it lacks one histidine residue (replaced by Lys70) essential for RNA degradation (Van Damme et al. [2000]). Therefore, the proteins encoded by AK061438 and AK109411 may be inactive for RNA degradation (MacIntosh et al. [2010]).

**Figure 4:**
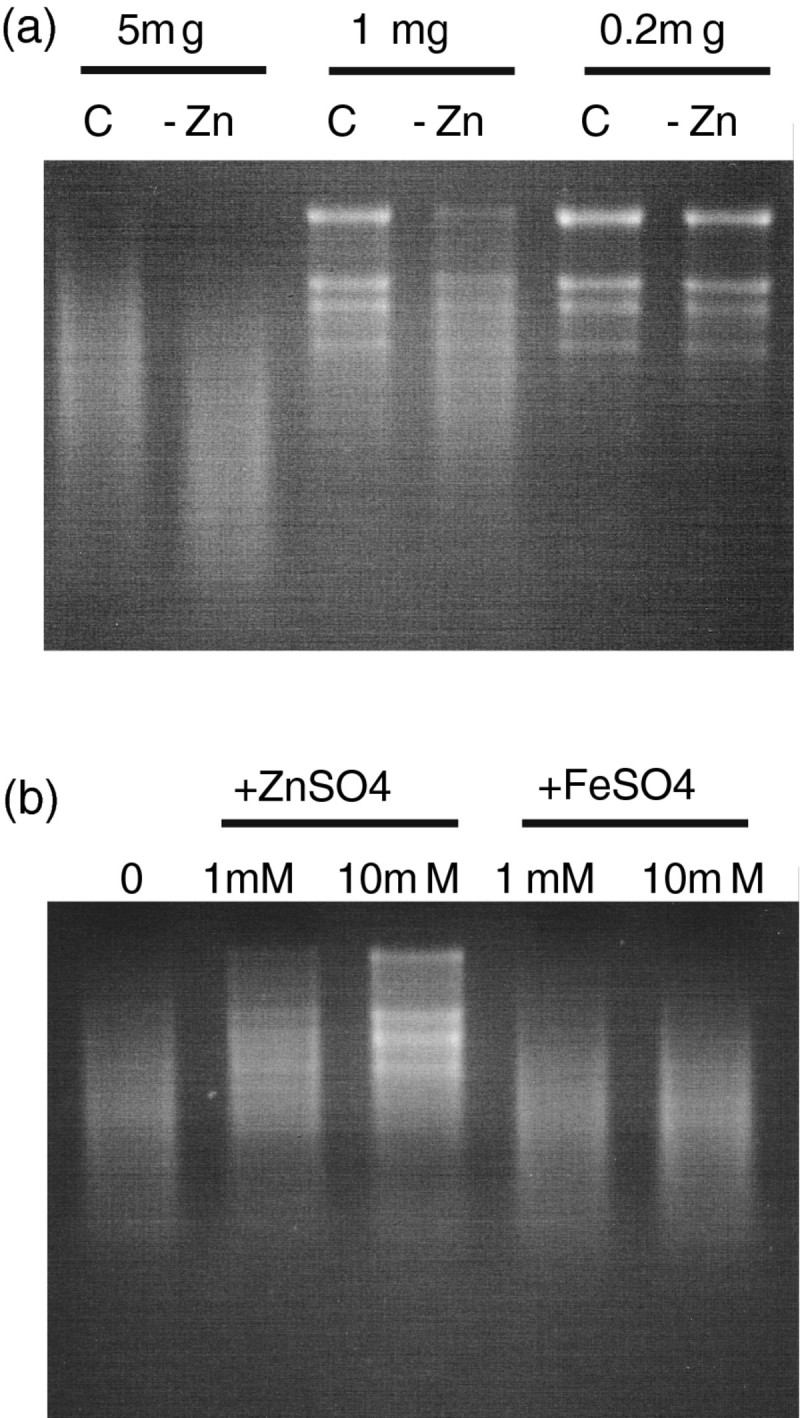
**RNAse activity in crude extracts of rice shoots. (a)** 10 μg of total RNA from Zn-deficient rice shoots were incubated with 5 μg, 1 μg, and 0.2 μg of protein from crude extracts of Zn-deficient shoots (−Zn) and control shoots (C). 3 μg of incubated total RNA were loaded into each lane. **(b)** 10 μg of total RNA from Zn-deficient rice shoots and 2 μg of protein from crude extracts of Zn-deficient shoots were incubated together with 1 mM or 10 mM ZnSO_4_ or FeSO_4_. 3 μg of incubated total RNA were loaded into each lane.

In contrast, RNA degradation increased in the crude extract of Zn-deficient citrullus (Sharma et al. [1981]) and black gram (Pandey et al. [2002]). RNA degradation in a crude extract of Zn-deficient rice shoots was also higher than that in control shoots (Figure 4a), and it was inhibited by adding ZnSO_4_ but not FeSO_4_ (Figure 4b). These findings agree with a previous report showing that Zn ions inhibited RNA degradation in a crude extract of avena leaves (Wyen et al. [1971]).

## Discussion

The expression of genes involved directly or indirectly in Zn transport increases under Zn deficiency. Moreover, the genes involved in starch synthesis and transport were up-regulated by Zn deficiency. Both Zn-deficient roots and shoots accumulated starch (Figure 1), in line with the gene expression pattern (Table 2). Starch may be used as a carbon source; therefore, it is assumed that Zn-deficient plants synthesize starch to withstand temporary abiotic stress. However, it is also possible that disruption of glycolysis by Zn deficiency causes an over-accumulation of soluble sugar, which ultimately results in starch accumulation. It is already reported that the activity of FBP aldolase in the glycolysis pathway decreased under Zn-deficiency in the leaves of oat and clover, and suggested that one of the reasons for a growth defect in plants grown under Zn-deficient conditions is the breakdown of normal carbohydrate metabolism (Quinlan-Watson [1951]). The concentration of soluble sugar and starch increase in Zn-deficient bean shoots (Marschner & Cakmak [1989]). An increase in soluble sugar would cause osmotic stress. We speculate that the starch might be synthesized to avoid osmotic stress due to increased soluble sugar in cells under Zn deficiency. Up-regulation of the genes that encode soluble sugar transporters due to Zn-deficiency suggests that sugar transporters help distribute sugar to avoid osmotic stress (Table 2).

Zn-deficient plants have increased RNase activity (Sharma et al. [1981]; Pandey et al. [2002]). We showed that Zn-deficient rice shoots also have increased RNA degradation (Figure 4a). Our microarray analysis revealed that the expression of one S-like RNase increased under Zn deficiency, and the transcript levels were much higher than those of other RNases (Table 3). However, unexpectedly, this RNase protein seemed to have no enzymatic activity as the histidine and glutamate residues essential for RNA degradation were changed (Figure 3a, b; (MacIntosh et al. [2010]; Kurihara et al. [1996]; Van Damme et al. [2000])). Our microarray analysis also showed that no gene for active RNases were up-regulated by Zn deficiency. In addition, Zn ions inhibited the RNase activity in a crude extract from Zn-deficient shoots (Figure 4b). These findings suggest that RNA degradation may be controlled by the Zn concentration at a cellular level rather than by the level of RNase mRNA.

The function of the inactive RNase induced by Zn deficiency is not clear. An inactive S-like RNase accumulate in the rhizomes of *Calystegia sepium* and is suggested to play a role in vegetative storage proteins (Van Damme et al. [2000]). Thus, Zn-deficient rice might accumulate inactive RNase for storage of amino acids. The inactive RNase, also, accumulate in response to drought stress (Salekdeh et al. [2002]), one of the most fatal stresses for plants. Therefore, the induction of the inactive RNase could be due to fatal stress for plants caused subsequently by Zn-deficiency stress. However, in our experiment, Fe-deficient stress decreased the expression of AK061438 and AK109411 (Figure 4), although Fe-deficient stress is more detrimental to plant growth than other micronutrient-deficient stresses. Moreover, the expression of AK061438 decreased under sulfur-deficient stress (Ohkama-Ohtsu et al. [2004]). These data suggest that the induction of putative inactive RNases is specifically caused by Zn-or water-deficient stress rather than by general stress conditions in plants.

Taken together, Zn-deficient rice plants accumulate starch that can be used as carbon and nitrogen sources. This might be involved in the tolerance to Zn deficiency, damage by Zn-deficient stress, or Zn homeostasis. To better understand these mechanisms, it is important to elucidate the differences between Zn-efficient and Zn-inefficient cultivars, and the molecular functions of each gene involved in tolerance to Zn deficiency should be revealed.

## Conclusions

Microarray analysis of Zn deficiency rice revealed the up-regulation of several genes involved in starch synthesis in Zn deficient rice roots and shoots. The accumulation of starch granules in the cortical cells of these tissues further supported these results. A gene encoding inactive RNase was much more highly transcribed than those encoding active RNases. Moreover, the level of RNA degradation in a crude extract of Zn-deficient shoots reduced after addition of Zn to a crude extract of Zn-deficient shoots. These results suggest that the tolerance of rice plants to low levels of Zn may be promoted by the accumulation of starch and inactive RNase proteins.

## Methods

### Plant materials and growth conditions

Rice seeds (*Oryza sativa* L.) were germinated for 1 week at room temperature on paper towels soaked with distilled water. After germination, the seedlings were transferred to a Saran net floating on nutrient solution in a glasshouse for 2 weeks. Two week old plants were transferred to a 20-L plastic container containing a nutrient solution as described previously (Suzuki et al. [2006]) with or without ZnSO_4_, The pH of the nutrient solution was adjusted daily to 5.5, and was renewed weekly. To compare the response of rice to Zn deficiency with that to deficiencies in other micronutrients, rice seeds were germinated for 1 week and then transferred to the nutrient solution described above. After 1 week, the seedlings were transferred to fresh nutrient solution without Zn, Fe, Cu or Mn and grown for 2 additional weeks.

### Microarray analysis

Roots and shoots of four week old plants subjected to Zn deficiency or grown under control conditions were collected, frozen in liquid nitrogen, and stored at −80°C until use. RNA was extracted from the roots and shoots of three plants, and total RNA (200 ng) from the Zn deficient and Zn sufficient plants were labelled with Cy3 or Cy5 using an Agilent Low RNA Input Fluorescent Linear Amplification Kit (Agilent Technologies), and rice 44 K oligo-DNA microarray analysis was performed in duplicate using color swaps, according to the manufacturer’s instructions, as described previously (Bashir et al. [2011]; Ishimaru et al. [2007]). The induction ratios shown in the tables were calculated as the relative increases in expression under Zn deficiency compared to the level of expression under control conditions.

### Quantification of starch and iodine staining

Rice seeds were germinated for 1 week and transferred to nutrient solution containing Zn. After 2 weeks, the seedlings were transferred to a Zn-deficient nutrient solution for 3 weeks and harvested for analysis. The samples were collected 2 h before sunset. To determine the level of starch, each plant was ground using a mortar and pestle with liquid nitrogen and dried overnight at 65°C. Around 50 mg of dried plant tissue was used for starch quantification, employing a total starch assay procedure kit (Megazyme, Bray, Ireland). To determine the localization of the starch granules, leaf blades and roots were cut with a scalpel into approximately 1-cm sections. These sections were embedded in 5% agar and then cut into 80- to 130-μm sections using a DTK-100 microslicer (Dosaka EM Co. Ltd., Kyoto, Japan) and stained with iodine. To remove the pigment in the leaf, the plant sections were soaked in 80% ethanol for 1 month.

### Measuring RNA degradation

RNA degradation was detected by electrophoresis (Van Damme et al. [2000]). Total RNA from rice shoots grown under Zn-deficient treatment for 3 weeks was used as a substrate. A 10-μg sample of total RNA was incubated in 20 μL of 25 M Tris–HCl (pH 7.4) containing 25 mM KCl and 5 mM MgCl_2_ in the presence of crude extracts from Zn-deficient and control shoots at 30°C for 15 min. To investigate the effects of metal on RNA degradation, 10 μg of RNA were incubated with 1 mM and 10 mM ZnSO_4_ and FeSO_4_ under the conditions described above. A 3-μg aliquot of incubated RNA diluted with distilled water was loaded onto a 1.2% agarose gel containing ethidium bromide. The extracts were prepared from 0.1 g of ground tissue mixed with 1 mL of Tris buffer. The amount of protein in the crude extract was quantified by the Bradford method.

### Northern blot analysis

Total RNA was extracted from roots and shoots, and 10 μg per lane were electrophoresed in 1.2% (w/v) agarose gels containing 0.66 M formaldehyde, transferred to Hybond-N^+^ membrane (Amersham Biosciences UK Ltd., Buckinghamshire, UK), and hybridized with probes at 65°C. Northern blots were analyzed using BAS 3000 (FujiFilm, Tokyo, Japan). The full-length rice cDNAs (Rice Genome Resource Center, Tsukuba, Japan) were used for labelling the probes.

## Authors’ information

**M.S.**; Present address; Aichi Steel Corporation, 1, Wanowari, Arao-Machi, Tokai-Shi, Aichi 476–8666, Japan **H.I.**; Present address: Division of Plant Sciences, National Institute of Agrobiological Sciences, 2-1-2 Kannondai, Tsukuba, Ibaraki 305–8602, Japan. **M.T.**; Present address: Faculty of Agriculture, Utsunomiya University - 350 Mine, Utsunomioya, Tochigi 321–8505, Japan.

## Electronic supplementary material

Additional file 1: Table-S1.Microarray analysis of genes involved in carbohydrate metabolism and transport in rice. The values present the average ± SE (n = 4). Two weeks old rice plants were subjected to Zn deficiency for two more weeks. **Table S2.** Microarray profile of genes encoding RNase in rice. The values present the average ± SE (n = 4). Two weeks old rice plants were subjected to Zn deficiency for two more weeks. (DOC 62 KB)

Below are the links to the authors’ original submitted files for images.

Authors’ original file for figure 1

Authors’ original file for figure 2

Authors’ original file for figure 3

Authors’ original file for figure 4

## Abbreviations

AGPase: ADP-glucose pyrophosphorylase
FBP aldolase: Fructose-1,6-bisphosphate aldolase.
