# Supplementary material for: Accumulation of starch in Zn-deficient rice
Source: Rice (N Y). 2012 Apr 6;5:9. doi: 10.1186/1939-8433-5-9 (PMC5520845; doi:10.1186/1939-8433-5-9)
Supplement: Supplementary file 1 — Additional file 1: Table-S1.Microarray analysis of genes involved in carbohydrate metabolism and transport in rice. The values present the average ± SE (n = 4). Two weeks old rice plants were subjected to Zn deficiency for two more weeks. Table S2. Microarray profile of genes encoding RNase in rice. The values present the average ± SE (n = 4). Two weeks old rice plants were subjected to Zn deficiency for two more weeks. (DOC 62 KB) [file 12284_2012_11_MOESM1_ESM.doc]

**Supplemetaty Table-1.** Microarray analysis of genes involved in carbohydrate metabolism and transport in rice. The values present the average ± SE (n = 4). Two weeks old rice plants were subjected to Zn deficiency for two more weeks.

| Accession No. | Putative gene identification | Ratio (-Zn/+Zn) | |
| --- | --- | --- | --- |
|  |  | root | shoot |
| starch metabolism | | | |
| AK109458 | *starch synthase* | 5.1 ± 0.5 | 5.3 ± 0.2 |
| AK102058 | *starch synthase* | 4.6 ± 0.4 | 5.5 ± 0.8 |
| AK100910 | *ADPGase large subunit* | 6.3 ± 0.6 | 7.3 ± 0.9 |
| AK073146 | *ADPGase small subunit* | 10.6 ± 1.0 | 6.0 ± 0.6 |
| AK068920 | *α-1,4-glucan branching enzyme* | 2.2 ± 0.2 | 4.2 ± 0.4 |
| AK065121 | *α-1,4-glucan branching enzyme* | 1.6 ± 0.1 | 3.6 ± 0.4 |
| AK064893 | *phosphoglucomtase* | 3.2 ± 0.2 | 2.2 ± 0.2 |
| AK099406 | *sucrose synthase* | 1.9 ± 0.1 | 2.4 ± 0.1 |
| AK102158 | *sucrose synthase* | 1.5 ± 0.1 | 2.1 ± 0.1 |
| AK063766 | *α-1,4-glucan phosphorylase* | 4.9 ± 0.4 | 5.6 ± 0.4 |
| AK103367 | *α-1,4-glucan phosphorylase* | 6.2 ± 0.1 | 2.1 ± 0.3 |
|  |  |  |  |
| glycolysis pathway | | | |
| AK068061 | *glucose-6-phosphate isomerase* | 1.2 ± 0.1 | 2.6 ± 0.3 |
| AK072857 | *Phosphofructokinase* | 1.2 ± 0.0 | 2.5 ± 0.0 |
| AK099939 | *Phosphofructokinase* | 1.3 ± 0.2 | 2.3 ± 0.5 |
| AK064960 | *glyceraldehyde 3-phosphate dehydrogenase* | 0.8 ± 0.0 | 2.2 ± 0.2 |
| AK100159 | *glyceraldehyde 3-phosphate dehydrogenase* | 2.0 ± 0.1 | 2.4 ± 0.3 |
| AK070705 | *phosphoglycerate kinase* | 0.9 ± 0.1 | 3.1 ± 0.2 |
| AK106501 | *phosphoglycerate kinase* | 0.9 ± 0.1 | 2.1 ± 0.1 |
| AK099387 | *fructose-bisphosphate aldolase (plastid)* | 0.9 ± 0.1 | 4.7 ± 1.0 |
| AK073758 | *fructose-bisphosphate aldolase (plastid)* | 2.6 ± 0.2 | 0.8 ± 0.1 |
| AK104952 | *fructose-bisphosphate aldolase (cytosolic)* | 2.4 ± 0.2 | 0.9 ± 0.1 |
|  |  |  |  |
| carbohydrate transport | | | |
| AK073967 | *hexose transporter* | 3.0 ± 0.3 | 2.9 ± 0.3 |
| AK073216 | *sugar transporter* | 2.0 ± 0.2 | 9.8 ± 2.8 |
| AK060577 | *glucose-6-phosphate translocator* | 1.5 ± 0.3 | 4.6 ± 1.3 |
| AK059423 | *sugar transporter* | 1.0 ± 0.1 | 4.9 ± 1.1 |
| AK063975 | *sugar carrier protein* | 1.0 ± 0.2 | 2.7 ± 0.1 |
| AK103047 | *sugar carrier protein* | 0.7 ± 0.1 | 2.2 ± 0.3 |
| AK099079 | *glucose transporter* | 0.7 ± 0.1 | 2.3 ± 0.3 |
| AK069202 | *glucose transporter* | 0.7 ± 0.1 | 3.5 ± 0.5 |

**Supplemetaty Table 2.** Microarray profile of genes encoding RNase in rice. The values present the average ± SE (n = 4). Two weeks old rice plants were subjected to Zn deficiency for two more weeks.

| Accession No. | Gene | Ratio (-Zn/+Zn) | | Putative  enzyme activity |
| --- | --- | --- | --- | --- |
|  |  | roots | shoots |  |
| AK060320 | *OsRNS1* | 0.4 ± 0.1 | 1.3 ± 0.3 | Yes |
| AK105061 | *OsRNS2* | 0.9 ± 0.1 | 1.4 ± 0.3 | Yes |
| AK058502 | *OsRNS3* | 0.8 ± 0.1 | 0.5 ± 0.0 | Yes |
| AK061438 | *OsRNS4* | 27.5 ± 3.2 | 6.1 ± 0.5 | **No** |
| AK109411 | *OsRNS5* | 1.0 ± 0.1 | 4.6 ± 1.7 | **No** |
| AK060320 | *OsRNS6* | 0.4 ± 0.1 | 1.3 ± 0.3 | Yes |
