# Supplementary figures and images for: Accumulation of starch in Zn-deficient rice
Source: Rice (N Y). 2012 Apr 6;5:9. doi: 10.1186/1939-8433-5-9 (PMC5520845; doi:10.1186/1939-8433-5-9)

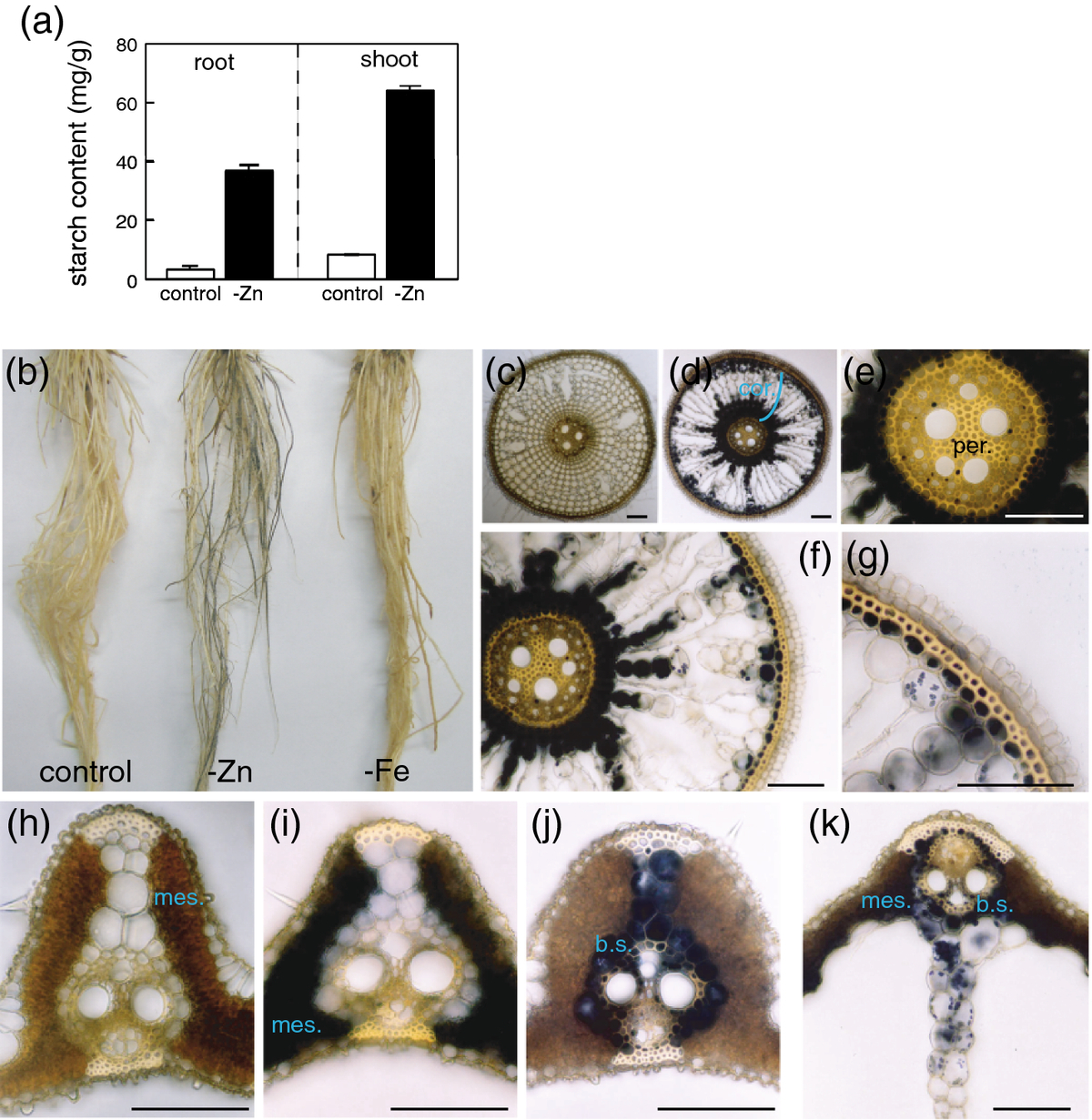

Supplement: Supplementary file 2 — Authors’ original file for figure 1 [file 12284_2012_11_MOESM2_ESM.jpeg]

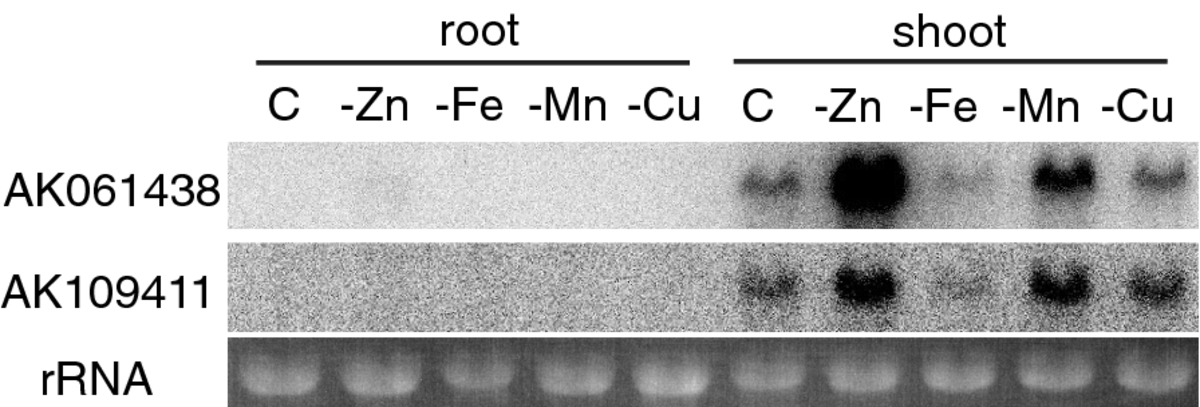

Supplement: Supplementary file 3 — Authors’ original file for figure 2 [file 12284_2012_11_MOESM3_ESM.jpeg]

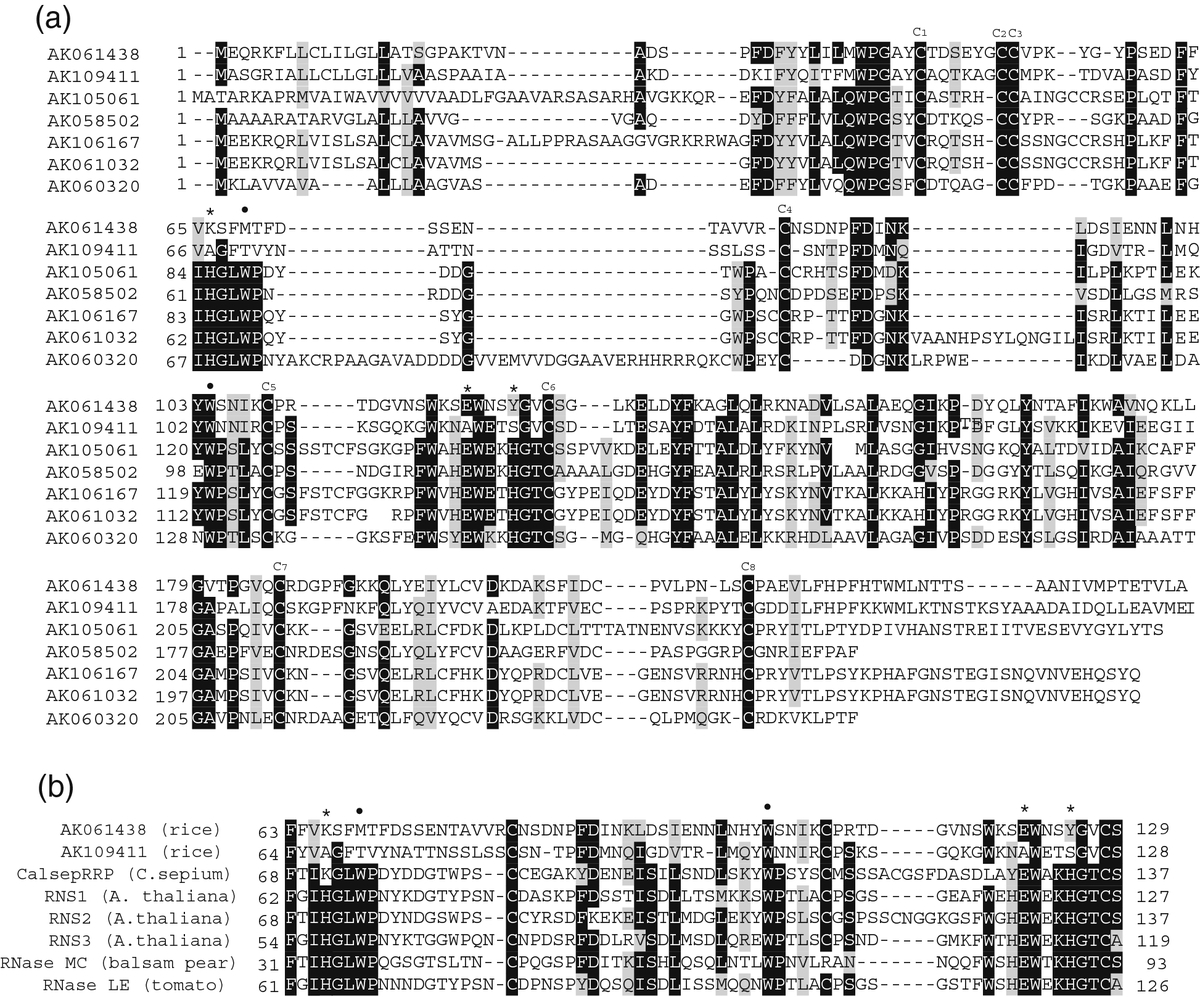

Supplement: Supplementary file 4 — Authors’ original file for figure 3 [file 12284_2012_11_MOESM4_ESM.jpeg]

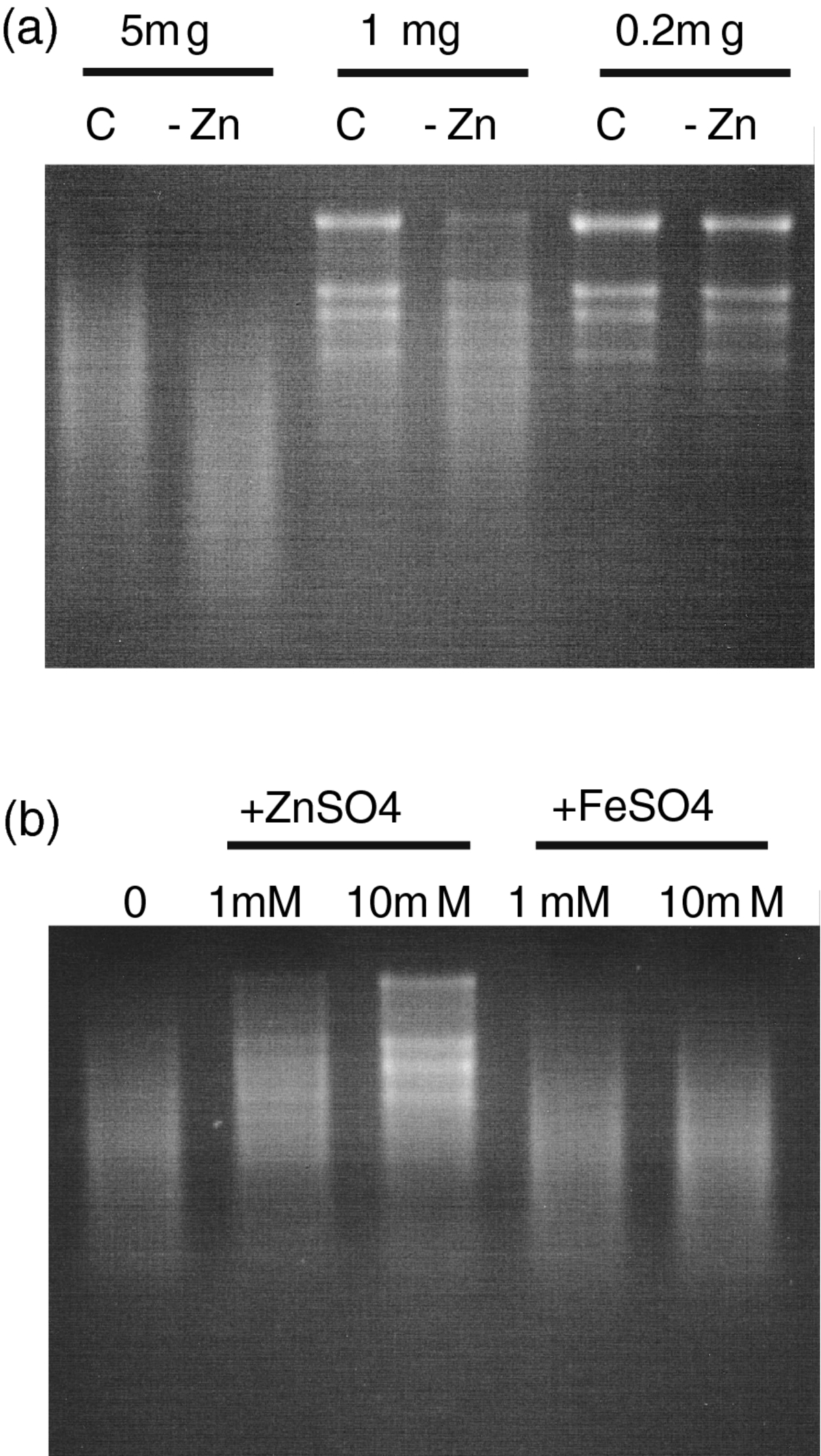

Supplement: Supplementary file 5 — Authors’ original file for figure 4 [file 12284_2012_11_MOESM5_ESM.jpeg]
